# Thrombomodulin phenotype of a distinct monocyte subtype is an independent prognostic marker for disseminated intravascular coagulation

**DOI:** 10.1186/cc10139

**Published:** 2011-04-14

**Authors:** Sang Mee Hwang, Ji-Eun Kim, Kyou-Sup Han, Hyun Kyung Kim

**Affiliations:** 1 Department of Laboratory Medicine, Seoul National University College of Medicine, 101, Daehak-ro Jongno-gu, Seoul 110-744, Republic of Korea; 2 Cancer Research Institute, Seoul National University College of Medicine, 101, Daehak-ro Jongno-gu, Seoul 110-744, Republic of Korea

## Abstract

**Introduction:**

Thrombomodulin, which is expressed solely on monocytes, along with tissue factor (TF), takes part in coagulation and inflammation. Circulating blood monocytes can be divided into 3 major subtypes on the basis of their receptor phenotype: classical (CD14^bright^CD16^negative^, CMs), inflammatory (CD14^bright^CD16^positive^; IMs), and dendritic cell-like (CD14^dim^CD16^positive ^DMs). Monocyte subtype is strongly regulated, and the balance may influence the clinical outcomes of disseminated intravascular coagulation (DIC). Therefore, we investigated the phenotypic difference in thrombomodulin and TF expression between different monocyte subtypes in coagulopathy severity and prognosis in patients suspected of having DIC.

**Methods:**

In total, 98 patients suspected of having DIC were enrolled. The subtypes of circulating monocytes were identified using CD14 and CD16 and the thrombomodulin and TF expression in each subtype, expressed as mean fluorescence intensity, was measured by flow cytometry. Plasma level of tissue factor was measured by ELISA. In cultures of microbead-selected, CD14-positive peripheral monocytes, lipopolysaccharide (LPS)- or interleukin-10-induced expression profiles were analyzed, using flow cytometry.

**Results:**

The proportion of monocyte subtypes did not significantly differ between the overt and non-overt DIC groups. The IM thrombomodulin expression level was prominent in the overt DIC group and was well correlated with other coagulation markers. Of note, IM thrombomodulin expression was found to be an independent prognostic marker in multivariate Cox regression analysis. In addition, *in vitro *culture of peripheral monocytes showed that LPS stimulation upregulated thrombomodulin expression and TF expression in distinct populations of monocytes.

**Conclusions:**

These findings suggest that the IM thrombomodulin phenotype is a potential independent prognostic marker for DIC, and that thrombomodulin-induced upregulation of monocytes is a vestige of the physiological defense mechanism against hypercoagulopathy.

## Introduction

Thrombomodulin (TM) is a transmembrane glycoprotein that blocks the interaction between thrombin and procoagulant protein substrates and acts as a vascular endothelial cell receptor for thrombin to activate protein C. Activated protein C inactivates factors Va and VIIIa and inhibits further thrombin generation and thus plays an important role in the anticoagulant state of the endothelium [1]. Tissue factor (TF) is an essential cofactor for the initiation of the extrinsic coagulation pathway. TF complexes with factors VII and VIIa and activates factors IX and X, and these activated factors contribute to the generation of thrombin on cell surfaces [2].

Disseminated intravascular coagulation (DIC) is characterized by systemic fibrin formation, resulting from increased generation of thrombin, simultaneous suppression of physiological anticoagulants, and impaired fibrinolysis [3]. A marked impairment in the protein C system worsens coagulopathy because the protein C pathway plays a role in the major regulatory loop that limits thrombin generation. This reduction in the protein C system is caused, in part, by the cytokine-induced decrement in TM activity and free protein S levels and impaired protein synthesis [3,4].

Monocytes play an important role in the coagulation system [5]. Endothelial cells and circulating monocytes express TF and TM within the vasculature [6]. Dysregulation of TF and TM expressions on cell surfaces may affect intravascular coagulation status. For example, inflammatory cytokines induce monocyte TF expression, which would yield procoagulant diathesis [5]. Also, in numerous pathophysiological conditions, monocyte TM expression was shown to be altered [7-9]. Therefore, one may speculate that the imbalance of the surface molecule expression of monocytes plays a role in the pathophysiology of DIC. In addition, monocytes, as key components of the humoral and cellular immune system, have been studied for subpopulation changes during infection and inflammatory conditions [10,11]. Whereas some inflammatory cytokines were known to increase TF of monocytes [12], anti-inflammatory cytokines such as IL-10 and IL-4 could suppress TF expression [13]. Because both inflammatory and anti-inflammatory cytokines are usually elevated in DIC, these cytokines may affect the expression of TF and TM in monocytes.

Monocytes subcategorized by the surface molecules CD14 and CD16 have been classified into three groups: CD14^bright^CD16^negative ^classical monocytes (CMs), which constitute the majority of circulating monocytes; CD14^bright^CD16^positive ^inflammatory monocytes (IMs), which produce proinflammatory cytokines; and CD14^dim^CD16^positive ^dendritic cell-like monocytes (DMs), which have features of differentiated monocytes or tissue macrophages, such as increased migration into tissues [14-16]. Many studies reported increases in the levels of IMs during inflammatory conditions such as in sepsis, rheumatoid arthritis, and hemolytic uremic syndrome [10,11,17]; however, changes in the DMs were variable [17-19].

In experimental models of sepsis, TF and TM mRNA upregulations through thrombin generation have been reported [7]. Monocyte subtype is strongly regulated, and the modulation of TF and TM expressions on monocyte subtype may influence the clinical outcomes of coagulopathy. Because the number of IMs are increased during inflammatory conditions [10], it can be hypothesized that the expression status of TF and TM on IMs may be a reflection of ongoing coagulopathy. Therefore, we investigated the phenotypic difference in TM and TF expressions among different monocyte subtypes associated with coagulopathy severity and prognosis in patients suspected of having DIC. Furthermore, to explore the changing pattern in expression phenotype of each monocyte subtype induced by both inflammatory stimuli and anti-inflammatory stimuli, the surface expression of TF and TM was investigated in monocytes derived from the *in vitro *culture of peripheral blood monocytes stimulated with lipopolysaccharide (LPS) and IL-10.

## Materials and methods

### Study population

A total of 98 patients who were clinically suspected of having DIC and who underwent screening battery tests of DIC were recruited for this study. This study was approved by the institutional review board of Seoul National University Hospital. Individual patient consent was not obtained, since all data used in this study were acquired retrospectively and anonymously from the laboratory information system without any additional blood sampling. Demographic and clinical data, including illness severity scores, were obtained from medical records (Table 1). Patients were labeled as having 'overt DIC' when their scores were at least 5 according to the International Society on Thrombosis and Haemostasis (ISTH) subcommittee scoring system [20,21]. Patients having a cumulative score of less than 5 were arbitrarily labeled as having 'non-overt DIC'.

**Table 1:** Characteristics of the study population

	Non-overt DIC	Overt DIC	Survivors	Non-survivors
Number	67	31	76	22
Age in years, mean (SD)	53.9 (17.4)	53.7 (12.6)	52.8 (16.8)	57.3 (12.7)
Gender, n (%)				
Male	40 (59.7)	21 (64.5)	46 (60.5)	15 (68.2)
Female	27 (40.3)	10 (35.5)	30 (39.5)	7 (31.8)
Clinical diagnosis, n (%)				
Sepsis/severe infection	10 (14.9)	8 (25.8)	11 (14.5)	7 (31.8)
Malignancies	21 (31.3)	12 (38.7)	22 (28.9)	10 (45.5)
Hepatic failure	14 (20.9)	11 (35.5)	23 (30.3)	2 (9.1)
Others^a^	22 (32.8)	0 (0.0)	19 (25.0)	3 (13.6)
SOFA score	3.0 (0.0-4.0)	7.0 (5.0-8.0)^b^	3.0 (0.0-5.0)	8.0 (5.0-8.8)^c^
SAPS II	22.0 (11.0-35.0)	44.0 (25.3-66.5)^b^	22.0 (12.0-37.0)	61.5 (27.5-74.5)^c^
Platelets, × 10^3^/μL	164.0 (60.0-236.0)	51.0 (33-67.5)^b^	133.5 (54.5-227.5)	56.5 (31.5-88.3)^c^
Prothrombin time, seconds	15.0 (13.7-15.9)	22.0 (19.5-24.3)^b^	15.0 (13.8-17.1)	21.6 (17.2-23.1)^c^
D-dimer, μg/mL	2.0 (0.9-4.6)	7.0 (4.6-12.4)^b^	2.0 (0.9-6.3)	5.5 (2.8-17.0)^c^
Fibrinogen, mg/dL	338 (260-451)	199 (127-272)^b^	303 (223-413)	272 (100-386)
Antithrombin, %	85 (60-112)	64 (32.5-81.5)^b^	79.5 (59-107.5)	54.5 (32.0-83.8)^c^
Protein C, %	67 (49-89)	27 (20-37.5)^b^	59.0 (38.5-85.3)	34.5 (22.0-73.5)^c^
Soluble tissue factor, pg/mL	68 (39-100)	98 (69-130)^b^	68.7 (41.1-96.8)	116.5 (93.2-138.1)^c^

Values are presented as median (interquartile range). ^a^'Others' refers to obstetric complications (*n *= 7), surgery (*n *= 6), aortic aneurysm (*n *= 3), and others (*n *= 6). ^b^*P *< 0.05 between non-overt disseminated intravascular coagulation (DIC) and overt DIC. ^c^*P *< 0.05 between 28-day survivors and 28-day non-survivors. SAPS II, Simplified Acute Physiology Score II; SD, standard deviation; SOFA, Sequential Organ Failure Assessment.

### Blood samples and plasma assays

Peripheral blood was collected in sodium citrate tubes (Becton, Dickinson and Company, Franklin Lakes, NJ, USA). The whole blood samples were centrifuged for 15 minutes at 1,550*g *within 2 hours of blood sampling. Prothrombin time (PT) and fibrinogen were assayed in accordance with a standard clotting assay on a STA-R analyzer (Diagnostica Stago, Asnières-sur-Seine, France). D-dimer was measured by immunoturbidimetric assay and protein C and antithrombin were measured by chromogenic assay on an ACL TOP (Beckman Coulter Inc., Fullerton, CA, USA). Plasma TF was measured with an Imubind Tissue Factor ELISA kit (American Diagnostica Inc., Stamford, CT, USA).

### Flow cytometric analysis

From ethylenediaminetetraacetic acid-treated whole blood that remained after measurement of complete blood cell count, peripheral blood mononuclear cells (PBMCs) were obtained by density gradient centrifugation over Ficoll-Paque (GE Healthcare Bio-Science AB, Uppsala, Sweden). Cell surface staining was performed on whole blood by using allophycocyanin-conjugated mouse anti-human CD14 (BD Biosciences, San Jose, CA, USA), fluorescein isothiocyanate-conjugated mouse anti-human CD16 (BD Biosciences), phycoerythrin-conjugated mouse anti-human tissue factor (BD Biosciences), and phycoerythrin-conjugated mouse anti-human TM (BD Biosciences). Appropriate isotype controls were used. On the basis of the scatter profile, monocytes were gated upon the lymphocyte tail on a FACSCalibur flow cytometer (Becton, Dickinson and Company, Franklin Lakes, NJ, USA). In total, 5,000 monocytes were acquired for each sample. Isotype-matched control antibodies were used to determine the cutoff between negative and positive CD14, CD16, TM, and TF. Once the monocyte population was evaluated with CD14 and CD16, each population was analyzed for the surface expression of TM and TF. Data were analyzed with FlowJo version 7.6.1 software (Tree Star, Inc., Ashland, OR, USA).

### *In vitro *phenotype of monocytes

Peripheral blood was collected from four healthy volunteers (one man and three women; mean age of 33.5 years) who provided informed consent. PBMCs were obtained by the above density gradient centrifugation method. Monocytes were purified from the PBMCs by using CD14 microbeads (Miltenyi Biotec Inc., Auburn, CA, USA) in accordance with the instructions of the manufacturer. More than 90% of the purified monocytes expressed surface CD14. The monocytes were suspended in RPMI 1640 medium containing 10% heat-inactivated fetal bovine serum (Invitrogen Corporation, Carlsbad, CA, USA) and stimulated with vehicle (phosphate-buffered saline), 100 ng/mL LPS (Sigma-Aldrich, St. Louis, MO, USA), or 10 ng/mL IL-10 (Pierce Endogen, Rockford, IL, USA). After 24 hours of incubation, the cells were stained for flow cytometric analysis.

### Statistical analysis

All statistical analyses were performed with SPSS 12.0 K for Windows (SPSS Inc., Chicago, IL, USA). Continuous data comparisons were performed by using the Mann-Whitney *U *rank sum test and Kruskal-Wallis tests, and the correlations were analyzed by using the Spearman's correlation coefficient. Comparison of categorical variables was performed by using the chi-square test. Kaplan-Meier survival analysis by the log-rank method was carried out for survival analysis of 28-day survival. Univariate and multivariate Cox regression analyses were performed to identify parameters to predict 28-day hospital mortality. The optimal cutoff values and diagnostic value of each parameter were determined with receiver operating characteristic (ROC) curve analysis by using MedCalc (MedCalc Software, Mariakerke, Belgium). A *P *value of less than 0.05 was set for statistical significance.

## Results

### Monocyte population according to overt disseminated intravascular coagulation status and mortality

Overt DIC status was diagnosed in 31 of 98 patients by using the ISTH diagnostic criteria (Table 1). There were no differences in age or gender between overt and non-overt DIC patients. Overt DIC patients showed lower platelet counts and fibrinogen, antithrombin, and protein C levels than non-overt DIC patients, and prothrombin time, D-dimer level, Sequential Organ Failure Assessment (SOFA) score, Simplified Acute Physiology Score II (SAPS II), and plasma TF level were significantly higher in the overt DIC patients. When divided into two groups by 28-day hospital mortality, clinical and laboratory parameters were also significantly different between the two groups.

The median percentage of monocyte subpopulation phenotype according to overt DIC status and mortality is shown in Table 2. The expression levels of TF and TM were significantly higher in IMs and DMs than in CMs in all patient groups (*P *< 0.001). The absolute monocyte count and the percentages of CMs, IMs, and DMs did not differ between the overt and non-overt DIC groups. In the overt DIC group, the TF expression level expressed by mean fluorescence intensity on CMs was lower than that in the non-overt DIC group, whereas the TM expression level of the IMs was significantly greater in the overt DIC group. The TF and TM expression levels of the DMs did not differ between the overt and non-overt DIC groups. In terms of hospital mortality, increased absolute monocyte count and increased expression of TM in the CMs were observed in the non-survival group. Of note, the markedly increased level of TM in the IMs was noted in the non-survival group. In addition, the TF and TM expressions on each monocyte subtype had positive correlations (CMs: *P *< 0.001, *r *= 0.497; IMs: *P *= 0.044, *r *= 0.205; DMs: *P *< 0.001, *r *= 0.362). However, there were no differences of TM and TF expressions on each monocyte subpopulation between the disease categories (data not shown).

**Table 2:** Percentage and phenotype of monocyte subpopulations according to overt disseminated intravascular coagulation status and mortality

		Non-overt DIC	Overt DIC	Survivors	Non-survivors
Number		67	31	76	22

Absolute monocyte count, × 10^6^/L		510 (336-752)	699 (351-1,260)	496 (337-743)	883 (452-1,913)^a^

CD14^bright^CD16^negative ^classic monocytes	Percentage	62.0 (48.3-70.9)	55.0 (48.4-65.6)	62.4 (51.1-70.7)	50.0 (39.2-54.5)^a^
	Thrombomodulin	32.0 (23.9-41.9)	29.0 (23.2-52.1)	31.1 (22.5-40.6)	35.9 (24.9-75.8)^a^
	Tissue factor	4.0 (3.4-4.5)	3.4 (2.6-4.4)^b^	4.0 (3.3-4.4)	3.5 (2.7-4.3)

CD14^bright^CD16^positive ^inflammatory monocytes	Percentage	13.0 (7.7-18.9)	11.0 (7.1-19.0)	12.8 (7.7-18.8)	10.7 (5.9-18.9)
	Thrombomodulin	55.0 (42.5-75.1)	70.0 (54.5-117.5)^b^	54.7 (43.1-71.9)	73.7 (60.5-125.5)^a^
	Tissue factor	5.4 (4.2-7.1)	5.6 (4.7-6.5)	5.5 (4.2-7.1)	5.3 (4.7-6.3)

CD14^dim^CD16^positive ^dendritic monocytes	Percentage	1.8 (0.8-4.4)	1.6 (1.0-3.2)	1.7 (0.8-3.6)	2.7 (1.0-6.2)
	Thrombomodulin	92.5 (49.9-114.8)	71.6 (47.7-115.0)	85.2 (46.7-114.5)	71.9 (55.2-115.8)
	Tissue factor	9.5 (5.1-20.7)	8.6 (6.1-16.2)	10.0 (5.2-19.4)	7.0 (5.9-17.0)

^a^*P *< 0.05 between survivors and non-survivors. ^b^*P *< 0.05 between non-overt disseminated intravascular coagulation (DIC) and overt DIC. The expression levels of thrombomodulin and tissue factor were scaled by an arbitrary unit of mean fluorescence intensity.

### Diagnostic performance of the thrombomodulin phenotype of the inflammatory monocytes

**Figure 1:**
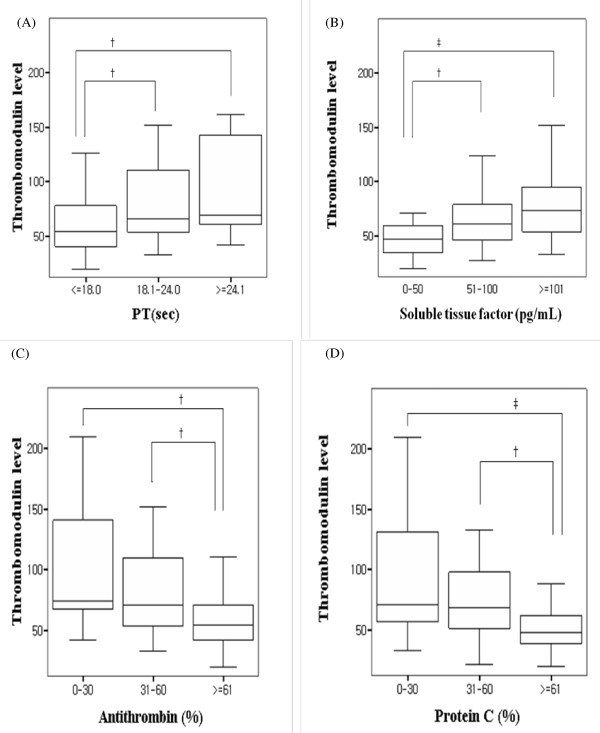
**Thrombomodulin expression level of inflammatory monocytes (CD14^bright^CD16^positive^)**. Levels are based on the prothrombin time (PT) (a) and plasma levels of tissue factor (b), antithrombin (c), and protein C (d). The expression level of thrombomodulin was scaled by an arbitrary unit of mean fluorescence intensity. The upper limit of each box represents the median value, and the bar represents the value of the 25th-75th percentile. ^†^*P *< 0.05, ^‡^*P *< 0.001.

Because the difference in the IM TM expression level between the overt and non-overt DIC groups was significant, we focused on the TM expression level of IMs as a potential marker of DIC. To investigate whether the IM TM level correlated with coagulopathy, we divided the patients into three tertile groups according to PT, TF, antithrombin, and protein C levels. Interestingly, the IM TM level gradually increased as PT and TF increased (Figure 1a, b). In addition, the IM TM level correlated with levels of both antithrombin and protein C (Figure 1c, d). In regard to the linear relationship between IM TM level and DIC markers, IM TM level was significantly correlated with PT (*P *< 0.001, *r *= 0.428), TF (*P *= 0.003, *r *= 0.307), antithrombin (*P *< 0.001, *r *= 0.451), and protein C (*P *< 0.001, *r *= -0.431) by Spearman's correlation analysis. The TM expression on IM was separately analyzed for the subgroups by disease categories. The correlation of TM expression on IM with coagulation markers was observed in the sepsis group with PT (*P = *0.009, *r *= 0.609), TF (*P *= 0.023, *r *= 0.565), antithrombin (*P *= 0.004, *r *= -0.662), and protein C (*P *= 0.010, *r *= -0.603). In the hepatic failure group, there was a correlation with PT (*P = *0.002, *r *= 0.580), antithrombin (*P *= 0.001, *r *= -0.606), and protein C (*P *= 0.002, *r *= -0.580). However, other subpopulations did not show correlations of TM expression on IM with coagulation markers individually.

**Figure 2:**
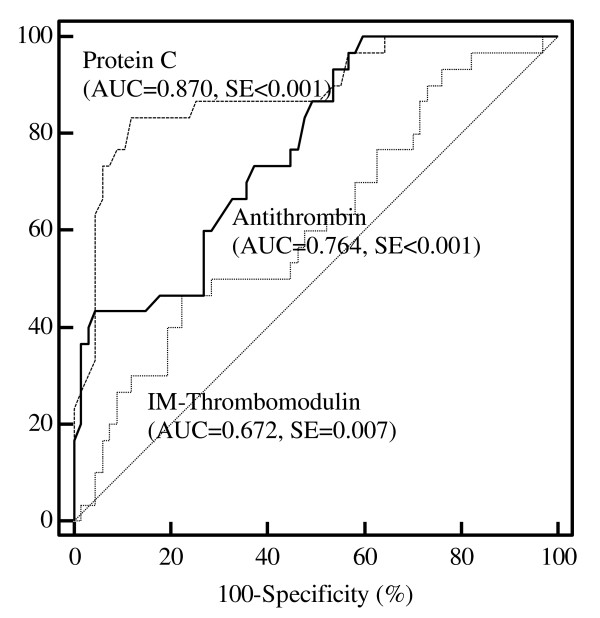
**Receiver operating characteristic (ROC) curves and the area under the ROC curves (AUC) for antithrombin, protein C, and thrombomodulin levels of CD14^bright^CD16^positive ^inflammatory monocytes (IM)**. Curves were used for the diagnosis of overt disseminated intravascular coagulation. SE, standard error.

The diagnostic value of IM TM level was evaluated by using the area under the ROC curve (AUC). The AUC of antithrombin and protein C, well-known DIC markers, showed significantly good discriminative power (Figure 2). The AUC of IM TM level was also significant but showed less discriminative power than that of antithrombin or protein C.

### Prognostic performance of the inflammatory monocyte thrombomodulin phenotype

**Figure 3:**
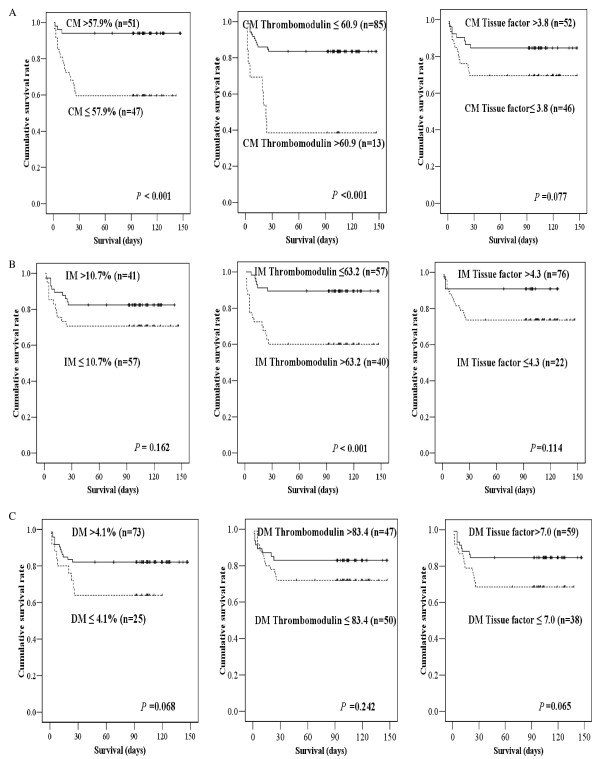
**Kaplan-Meier survival analysis according to proportions and expression levels of thrombomodulin and tissue factor**. Proportions and expression levels of (a) classical monocytes (CM), (b) inflammatory monocytes (IM), and (c) dendritic monocytes (DM) are shown. The cutoff values were determined as the values at which the prognostic power to predict 28-day mortality were the highest.

Twenty-eight-day hospital mortality was used as a parameter of clinical prognosis. The cutoff values of different markers for DIC were defined as the value at which the ROC curves showed optimal prognostic power. Patient groups with higher CM percentages (>57.9%) and lower TM expression levels of CMs (≤60.9) and IMs (≤63.2) showed better survival compared with those with lower CM percentages and higher TM expression levels of CMs and IMs (Figure 3). However, there were no significant differences in survival of the groups divided by the characteristics (the percentages or TM or TF expression) of DM.

Cox univariate analysis showed that decreased platelet count and prolonged PT, elevated D-dimer, low fibrinogen, low antithrombin, low protein C, and high plasma TF levels were significant predictors of 28-day mortality (Table 3). As for the monocyte phenotypes, high absolute monocyte count, low CM percentage, and high CM and IM TM expression were significant predictors for 28-day mortality in Cox univariate analysis. The TF expression levels of CM and IM were not statistically significant in univariate analysis, but in Cox multivariate analysis, low CM TF expression was an independent predictor of mortality along with fibrinogen and IM TM level.

**Table 3:** Univariate and multivariate analyses for predictors of 28-day mortality

	Univariate			Multivariate		
	
Variables	HR	95% CI	*P *value	HR	95% CI	*P *value
Platelet (>112 vs. ≤112 × 10^9^/L)	5.54	1.64-18.75	0.012	1.30	0.18-9.50	0.797
Prothrombin time (≤18.4 vs. >18.4 s)	7.25	2.94-17.85	<0.001	2.20	0.17-29.07	0.548
D-dimer (≤2.0 vs. >2.0 μg/mL)	8.57	2.00-36.69	0.004	3.48	0.45-27.11	0.233
Fibrinogen (>118 vs. ≤118 mg/dL)	7.35	2.92-18.48	<0.001	22.35	2.25-221.81	0.008
Antithrombin (>35% vs. ≤35%)	7.50	3.18-17.65	<0.001	2.15	0.13-36.70	0.598
Protein C (>27% vs. ≤27%)	4.04	1.74-9.37	0.001	1.63	0.06-47.58	0.777
Soluble tissue factor (≤106.1 vs. >106.1 pg/mL)	3.59	2.71-18.47	<0.001	1.20	1.73-8.36	0.852
Absolute monocyte count (≤755 vs. >755 × 10^6^/L)	3.76	1.61-8.81	0.002	2.31	0.39-13.71	0.359
CD14^bright^CD16^negative ^classical monocytes						
Percentage (>57.9% vs. ≤57.9%)	8.16	2.41-27.61	0.001	4.94	0.66-37.01	0.120
Thrombomodulin (≤60.9 vs. >60.9)	4.93	2.06-11.81	<0.001	1.36	0.36-5.18	0.649
Tissue factor (>3.8 vs. ≤3.8)	2.14	0.90-5.11	0.086	5.27	1.14-24.47	0.034
CD14^bright^CD16^positive ^inflammatory monocytes						
Percentage (≤10.7% vs. >10.7%)	1.80	0.78-4.17	0.171	1.36	0.25-7.25	0.722
Thrombomodulin (≤63.2 vs. >63.2)	4.67	1.82-11.94	0.001	19.11	1.51-241.47	0.023
Tissue factor (≤4.3 vs. >4.3)	3.03	0.71-12.98	0.135	1.36	0.07-25.34	0.836
CD14^dim^CD16^positive ^dendritic monocytes						
Percentage (>4.1% vs. ≤4.1%)	2.17	0.93-5.08	0.074	5.14	0.81-32.40	0.082
Thrombomodulin (>83.4 vs. ≤83.4)	1.67	0.40-3.98	0.249	1.12	0.13-9.85	0.918
Tissue factor (>7.0 vs. ≤7.0)	2.21	0.93-5.24	0.073	1.28	0.26-6.22	0.762

The cutoff values were determined as the values at which the best prognostic value was produced.

The expression levels of thrombomodulin and tissue factor were scaled by an arbitrary unit of mean fluorescence intensity. CI, confidence interval; HR, hazard ratio.

### Monocyte subtype proportion and expression phenotype patterns in an *in vitro *culture system

**Figure 4:**
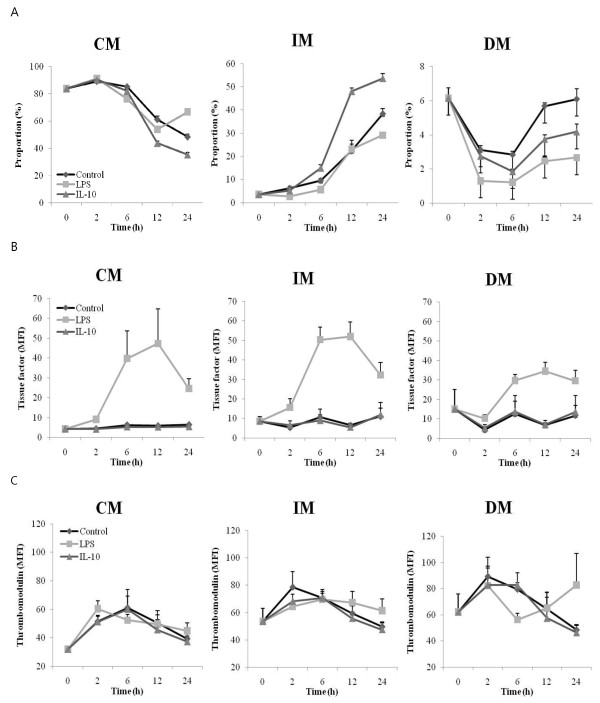
**Changes in the proportion and expression phenotype of a monocyte subtype cultured *in vitro***. Purified monocytes from healthy donors (*n *= 4) were cultured *in vitro *for 24 hours with vehicle, 100 mg/dL lipopolysaccharide (LPS), or 10 ng/mL interleukin-10 (IL-10). **(a) **Changes in the proportion and phenotype of **(b) **tissue factor and **(c) **thrombomodulin expression among three monocyte subtypes - classical monocytes (CM), inflammatory monocytes (IM), and dendritic monocytes (DM) - are shown over culture time. MFI, mean fluorescence intensity.

Purified monocytes from PBMCs of healthy donors were cultured *in vitro *for 24 hours. *In vitro *monocyte cultures showed decreasing CM and DM percentages and an increasing IM percentage (Figure 4). The IL-10-treated group revealed a further CM decrease and a corresponding IM increase compared with the control and LPS-treated groups (Figure 4a). The DM proportion decreased in the LPS- and IL-10-treated groups compared with the control group. The LPS-treated group showed markedly high TF expression in all monocyte subpopulations. The IL-10-treated group tended to exhibit slightly low TF expression, but the difference was not significant. TM expression levels increased the most in DMs, followed by IMs, and then finally CMs. In the LPS-treated group, CMs showed high TM expression at 2 hours, whereas IMs showed higher TM expression from 12 to 24 hours of culture in comparison with that of the control. In all monocyte subpopulations, IL-10 treatment tended to slightly decrease TM expression.

## Discussion

Tightly controlled TF and TM expressions maintain normal rheological properties of the blood. However, various stimuli such as infection and inflammation can induce inflammatory cytokines that increase TF expression and suppress anticoagulant protein expression [22-24]. This imbalance would eventually yield to the procoagulant diathesis of DIC. Therefore, the changed pattern of TF and TM expressions plays an important role in various pathophysiological conditions. Although the vascular endothelium is known to express TF and TM [6], circulating monocytes are also important cellular sources of TF and TM expressions within vessels [5]. The existence of different populations of monocytes (CMs, IMs, and DMs) is well established, and each population has a distinct antigen phenotype and function [11]. To date, there are no data on the expression pattern of TF and TM in any of these monocyte subpopulations. This study was the first to demonstrate the phenotypic changes of TF and TM in each monocyte subpopulation during DIC.

Interestingly, IM TM expression was prominent in the overt DIC group and had good correlation with other coagulation markers. Of note, IM TM expression was found to be an independent prognostic marker for DIC, which has been the focus of this study. Other phenotypic changes of the monocytes also showed differences between the overt and non-overt DIC, such as the lower TF expression of CMs in the overt DIC group. TF expression of CM was significant in multivariate analysis, but the correlations with other coagulation markers were weak and the differences between the survivor/non-survivor groups were minimal, and this needs to be studied further. When the survivors and non-survivors were compared, the percentage of CM was lower and TM expression on CMs and IMs was higher in the non-survivors. The TM expression on CM was significant in the univariate analysis but was not found to be an independent prognostic factor. In addition, the TM and TF expressions of DMs were higher than those of the IMs, but the mean differences of the TM and TF expressions of DMs between survivors and non-survivor were not significant and the phenotype of DMs was not found to be significant in multivariate analysis. These findings support the clinical relevance and importance of TM rather than TF expression in IMs.

Evaluation of the TF and TM expressions on each monocyte subtype showed positive correlation within each subpopulation of the monocytes. TF is a well-known initiator of coagulation and an important modulator of inflammation induced by proinflammatory cytokines [12], but the TM functions as both an anticoagulant and an anti-inflammatory molecule [25], so it is necessary to understand how TM expression is integrated to maintain homeostasis under hypercoagulable and proinflammatory conditions. TM is known to be transcriptionally upregulated by thrombin, vascular endothelial growth factor, histamine, dibutyryl cAMP, retinoic acid, theophylline, and statin, whereas shear stress, hemodynamic forces, hypoxia, and oxidized low-density lipoprotein suppress its expression [25]. In our study, TM expression tended to increase in hypercoagulable conditions. This finding is consistent with that of the previous *in vitro *experiment, which showed that viral stimulation increased TM expression in monocytes and endothelial cells [8]. This is also in agreement with the study that showed thrombin-induced upregulation of TM mRNA levels [7] and with the study that showed increased amounts of surface TM on monocytes during meningococcal disease [9]. All of these findings support the general notion that infection or inflammation shifts the hemostatic balance to thrombosis.

Although IM expansion was shown in inflammatory conditions [17-19], it is currently unclear how to change the TM phenotype of IMs. In our study, the IM TM expression level was highly associated with severe coagulopathy and poor prognosis, but those of CMs and DMs were not. This finding suggests that IMs play a role in maintaining the hemostatic balance of the active anticoagulant system by enhancing TM expression. The vivid reaction of IMs can be speculated from that of a previous study, which states that IMs produce proinflammatory cytokines [11]. The surface-bound TM is theoretically considered to be a regulator of the coagulation cascade in monocytes. However, it remains unclear whether IM TM expression exerts functional activity to dampen hypercoagulation. In our study, coagulopathy was severe in patients with high levels of TM, suggesting that the enhanced expression of TM in IMs plays an insufficient role in regulating the inflammatory sequelae. This change might just be the result of a physiological defense mechanism against hypercoagulopathy [26].

In our result, the percentage of monocyte subpopulations did not significantly differ between the overt and the non-overt DIC groups. Most related studies have compared the monocyte subpopulations between control and sepsis patients [17-19]. However, our study focused on patients suspected of having DIC (some with a recent inflammatory insult, others with overlaying stimuli in chronic conditions, and others in recovery); thus, the result may not show a clear-cut difference between the overt and the non-overt groups. This heterogeneity within each subgroup may have created a less dramatic difference between the expression level of TF or TM on monocytes as well.

To evaluate the diagnostic value of the IM TM phenotype, we analyzed the AUC value and compared it with that of well-known DIC markers. The AUC for the TM phenotype was significant (0.672) but was lower than that of protein C and antithrombin, suggesting that the IM TM phenotype is not a good diagnostic marker of overt DIC. On the other hand, it was useful for estimating prognosis. IM TM expression remained a significant prognostic factor in multivariate Cox analysis, with a hazard ratio of 19.11 after adjustment for the effect of other coagulation markers. Given that most of the DIC markers are dependent on each other, the IM TM phenotype is expected to be a useful potential marker of prognosis. A future prospective study is needed to verify the prognostic value of this marker.

*In vitro *culture results showed that the IM proportion increased with culture time in both control and stimulated monocytes. Interestingly, IL-10 induced a high proportion of IMs and a correspondingly low proportion of CMs in comparison with LPS or no treatment. Moreover, IL-10 treatment tended to decrease TF and increase TM, although the difference was minimal. Given that IL-10 is an anti-inflammatory cytokine, these actions are thought to be counter-responsive to the inflammatory stimuli. Our suggestion is in good agreement with a previous report in which the alternative activation of monocytes by IL-10 induced a phenotype that promoted tissue repair and suppressed inflammation [14]. On the other hand, TF expression in all monocyte subpopulations increased in the LPS-treated group, as observed in other studies [13,24,27]. An elegant study reported that TF mRNA levels in leukocytes increased during DIC [28]. In our clinical results, TF expression was not a significant marker except in CM, in which low TF expression predicted poor prognosis. It is currently unclear why low TF expression represents poor prognosis. In our data, the TF expression between overt and non-overt DIC was not different, although *in vitro *culture suggested that LPS induced the expression of both TF and TM. In the *in vitro *experiment, monocytes from healthy individuals were stimulated with an inflammatory stimulus (LPS), reflecting the basic modulation of TF and TM expressions by an inflammatory insult. However, the studied population is a heterogeneous group even in the overt or non-overt DIC group; thus, the result may not show a clear-cut difference between the overt and the non-overt groups. TM expression did not differ significantly between the three monocyte subpopulations, but LPS treatment upregulated TM at 2 hours in CMs and at 12 to 24 hours in IMs. We [29] and another group [30] previously reported that LPS downregulated TM expression in monocytes. However, we could not demonstrate LPS-induced TM downregulation. We speculate that the difference in expression may be a result of different culture conditions. Previous experiments used a culture of PBMCs that included high numbers of lymphocytes [29,30], and this potentially produces amounts of inflammatory cytokines that can affect the TM level. In this experiment, we used purified monocytes that contained low numbers of lymphocytes. Upregulation of TM may contribute to the regulation of coagulation by promoting activated protein C, thus suggesting a defense mechanism against the development of extensive microvascular fibrin deposition during DIC. However, as shown in our clinical study, insufficient TM function is expected in monocytes.

## Conclusions

The peripheral monocytes of patients suspected of having DIC were categorized into three subtypes and studied for TM and TF expressions. The IM TM expression level showed a significant correlation with the known DIC markers and had diagnostic value for overt DIC. Furthermore, the IM TM expression level was found to be an independent prognostic factor for 28-day mortality in DIC. In addition, *in vitro *culture of peripheral monocytes showed that LPS stimulation upregulated TM and TF expressions in a distinct subtype of monocytes. These findings suggest that IM TM upregulation is a vestige of the physiological defense mechanism against hypercoagulopathy and is a good potential independent prognostic marker for DIC.

## Key messages

• Thrombomodulin expression level of inflammatory monocytes shows a significant correlation with the known disseminated intravascular coagulation (DIC) markers and had diagnostic value for overt DIC.

• Thrombomodulin expression of inflammatory monocytes is an independent prognostic marker in patients suspected of having DIC.

• Lipopolysaccharide stimulation upregulates thrombomodulin and tissue factor expression in a distinct subtype of monocytes in *in vitro *culture of peripheral monocytes.

## Abbreviations

AUC: area under the receiver operating characteristics curve; CM: classical monocyte; DIC: disseminated intravascular coagulation; DM: dendritic cell-like monocyte; IL: interleukin; IM: inflammatory monocyte; ISTH: International Society on Thrombosis and Haemostasis; LPS: lipopolysaccharide; PBMC: peripheral blood mononuclear cell; PT: prothrombin time; ROC: receiver operating characteristic; TF: tissue factor; TM: thrombomodulin.

## Competing interests

The authors declare that they have no competing interests.

## Authors' contributions

HKK designed the study, shared responsibility for the study design and for data management and statistical analysis, and helped to write the manuscript. JEK performed the experiments and shared responsibility for data management and statistical analysis. SMH shared responsibility for data management and statistical analysis and helped to write the manuscript. KSH shared responsibility for the study design, data interpretation, and manuscript revision for important intellectual content. All authors read and approved the final manuscript.

## References

[B1] Sarangi P Lee H Kim M Activated protein C action in inflammation Brit J Haematol 2010 148 817 833 10.1111/j.1365-2141.2009.08020.x PMC2868910 19995397

[B2] Persson E Olsen O Current status on tissue factor activation of factor VIIa Thromb Res 2010 125 S11 S12 20153879 10.1016/j.thromres.2010.01.023

[B3] Levi M Ten Cate H Disseminated intravascular coagulation N Engl J Med 1999 341 586 592 10.1056/NEJM199908193410807 10451465

[B4] Conway EM Rosenberg RD Tumor necrosis factor suppresses transcription of the thrombomodulin gene in endothelial cells Mol Cell Biol 1988 8 5588 5592 2854203 10.1128/mcb.8.12.5588-5592.1988 PMC365667

[B5] Moore K Andreoli S Esmon N Esmon C Bang N Endotoxin enhances tissue factor and suppresses thrombomodulin expression of human vascular endothelium in vitro J Clin Invest 1987 79 124 130 10.1172/JCI112772 3025256 PMC424004

[B6] McCachren S Diggs J Weinberg J Dittman W Thrombomodulin expression by human blood monocytes and by human synovial tissue lining macrophages Blood 1991 78 3128 3132 1660324

[B7] Bartha K Brisson C Archipoff G de la Salle C Lanza F Cazenave J Beretz A Thrombin regulates tissue factor and thrombomodulin mRNA levels and activities in human saphenous vein endothelial cells by distinct mechanisms J Biol Chem 1993 268 421 429 7678000

[B8] Chen LC Shyu HW Lin HM Lei HY Lin YS Liu HS Yeh TM Dengue virus induces thrombomodulin expression in human endothelial cells and monocytes in vitro J Infection 2009 58 368 374 10.1016/j.jinf.2009.02.018 19307023

[B9] Faust S Heyderman R Levin M Coagulation in severe sepsis: a central role for thrombomodulin and activated protein C Crit Care Med 2001 29 S62 67 11445736 10.1097/00003246-200107001-00022

[B10] Fingerle G Pforte A Passlick B Blumenstein M Strobel M Ziegler-Heitbrock H The novel subset of CD14+/CD16+ blood monocytes is expanded in sepsis patients Blood 1993 82 3170 3176 7693040

[B11] Ziegler-Heitbrock L The CD14+ CD16+ blood monocytes: their role in infection and inflammation J Leukocyte Biol 2007 81 584 592 17135573 10.1189/jlb.0806510

[B12] Levi M van der Poll T ten Cate H Tissue factor in infection and severe inflammation Semin Thromb Hemost 2006 32 33 39 10.1055/s-2006-933338 16479460

[B13] Lindmark T Chen S IL-10 inhibits LPS-induced human monocyte tissue factor expression in whole blood Brit J Haematol 1998 102 597 604 10.1046/j.1365-2141.1998.00808.x 9695979

[B14] Gordon S Taylor P Monocyte and macrophage heterogeneity Nat Rev Immunol 2005 5 953 964 10.1038/nri1733 16322748

[B15] McPherson R Pincus M Henry's Clinical Diagnosis and Management by Laboratory Methods 2006 21 Philadelphia: Saunders21527533

[B16] Thomas R Lipsky P Human peripheral blood dendritic cell subsets. Isolation and characterization of precursor and mature antigen-presenting cells J Immunol 1994 153 4016 4028 7523513

[B17] Skrzeczynska J Kobylarz K Hartwich Z Zembala M Pryjma J CD14+ CD16+ monocytes in the course of sepsis in neonates and small children: monitoring and functional studies Scand J Immunol 2002 55 629 638 10.1046/j.1365-3083.2002.01092.x 12028567

[B18] Poehlmann H Schefold J Zuckermann-Becker H Volk H Meisel C Phenotype changes and impaired function of dendritic cell subsets in patients with sepsis: a prospective observational analysis Crit Care 2009 13 R119 10.1186/cc7969 19604380 PMC2750167

[B19] Skinner N MacIsaac C Hamilton J Visvanathan K Regulation of Toll like receptor (TLR) 2 and TLR4 on CD14dimCD16+ monocytes in response to sepsis related antigens Clin Exp Immunol 2005 141 270 278 10.1111/j.1365-2249.2005.02839.x 15996191 PMC1809439

[B20] Toh C Hoots W The scoring system of the Scientific and Standardisation Committee on Disseminated Intravascular Coagulation of the International Society on Thrombosis and Haemostasis: a 5-year overview J Thromb Haemost 2007 5 604 606 10.1111/j.1538-7836.2007.02313.x 17096704

[B21] Taylor F Toh C Hoots W Wada H Levi M Towards definition, clinical and laboratory criteria, and a scoring system for disseminated intravascular coagulation Thromb Haemost 2001 86 1327 1330 11816725

[B22] Osterud B Bjorklid E The tissue factor pathway in disseminated intravascular coagulation Semin Thromb Haemost 2001 27 605 618 10.1055/s-2001-18866 11740684

[B23] Celi A Pellegrini G Lorenzet R De Blasi A Ready N Furie B Furie B P-selectin induces the expression of tissue factor on monocytes Proc Natl Acad Sci USA 1994 91 8767 8771 10.1073/pnas.91.19.8767 7522321 PMC44687

[B24] Osterud B Tissue factor expression in monocytes: in vitro compared to ex vivo Thromb Haemostasis 2000 84 521 522 11019986

[B25] Van de Wouwer M Collen D Conway EM Thrombomodulin-protein C-EPCR system: integrated to regulate coagulation and inflammation Arterioscl Thromb Vas 2004 24 1374 1383 10.1161/01.ATV.0000134298.25489.92 15178554

[B26] Tsai C Tsai Y Lin C Lin T Huang G Hong G Lin F Expression of thrombomodulin on monocytes is associated with early outcomes in patients with coronary artery bypass graft surgery Shock 2010 34 31 39 20090566 10.1097/SHK.0b013e3181d494c4

[B27] Herbert J Savi P Laplace M Lale A IL-4 inhibits LPS-, IL-1 [beta]-and TNF [alpha]-induced expression of tissue factor in endothelial cells and monocytes FEBS Lett 1992 310 31 33 10.1016/0014-5793(92)81139-D 1526281

[B28] Sase T Wada H Nishioka J Abe Y Gabazza EC Shiku H Suzuki K Nakamura S Nobori T Measurement of tissue factor messenger RNA levels in leukocytes from patients in hypercoagulable state caused by several underlying diseases Thromb Haemost 2003 89 660 665 12669120

[B29] Kim H Kim J Chung J Kim Y Kang S Han K Cho H Lipopolysaccharide down-regulates the thrombomodulin expression of peripheral blood monocytes: effect of serum on thrombomodulin expression in the THP-1 monocytic cell line Blood Coagul Fibrin 2007 18 157 164 10.1097/MBC.0b013e32801481cb 17287633

[B30] Satta N Freyssinet J Toti F The significance of human monocyte thrombomodulin during membrane vesiculation and after stimulation by lipopolysaccharide Brit J Haematol 1997 96 534 542 10.1046/j.1365-2141.1997.d01-2059.x 9054661

